# Hospital-based descriptive epidemiology of intracranial meningiomas in Palestine: A retrospective study from a major referral center (2018–2023)

**DOI:** 10.1371/journal.pone.0339114

**Published:** 2026-07-06

**Authors:** Mohammad Abuawad, Ahmad Rjoub, Yazan Dumaidi, Motaz Daraghma, Mohammad A. Nour, Wafaa Abu Zahra, Noor Issa, Ahmed Daqour, Abdulsalam Alkaiyat

**Affiliations:** 1 Department of Biomedical Sciences and Basic Clinical Skills, Faculty of Medicine and Allied Medical Sciences, An-Najah National University, Nablus, Palestine; 2 Department of Medicine, Faculty of Medicine and Allied Medical Sciences, An-Najah National University, Nablus, Palestine; 3 Faculty of Medicine, Arab American University of Palestine, Jenin, Palestine; 4 Department of Neurosurgery, Almakassed Hospital, East Jerusalem, Palestine; 5 Department of Medicine, Al-Quds University, East Jerusalem, Palestine; West Bengal State University, INDIA

## Abstract

**Objective:**

Intracranial meningioma is the most common type of primary brain tumor globally. This study aimed to determine the epidemiological characteristics of intracranial meningioma in Palestine from 2018 to 2023.

**Methods:**

We conducted a retrospective hospital-based descriptive study of radiologically and histologically confirmed intracranial meningiomas treated or referred to Al-Makassed Hospital, the main neurosurgical referral center in Palestine, from September 7^th^, 2023, to November 16^th^, 2023. Patients’ reports were the main source for data collection. Histopathological types of meningioma were coded according to the 2016 WHO Classification for CNS tumors. The incidence rates of intracranial meningiomas were hospital-based referral-center minimum estimates, calculated based on the total population size.

**Results:**

A total of 204 cases of intracranial meningioma were identified. The mean age at diagnosis was 51.12 years (±13.96). The estimated minimum hospital-based crude incidence rate was 0.65 per 100,000 person-years (95% CI: 0.56–0.75), with a higher incidence in females than in males (0.88 vs 0.43, (0.88 vs 0.43 per 100,000 person-years, IRR = 2.07 [95% CI: 1.53–2.81]; p = 0.003). Grade I meningiomas were the most common, accounting for 81.4%. Grade II and III meningiomas were more common in males. Focal Neurological Symptoms (39.7%) were the most prevalent presenting symptoms. Intracranial meningiomas were most often managed with surgical resection alone (96.6%).

**Conclusion:**

This study provided insights into the epidemiological characteristics of meningioma in Palestine. These findings represent hospital-based minimum estimates and are not directly comparable to population-based registry data. Meningiomas were more common in females, while higher-grade meningiomas were more common in males. Further research is needed to ascertain the most common subtypes.

## 1 Introduction

Meningiomas are tumors that originate from the meninges, the protective membranes that cover the brain and spinal cord. They are slow-growing, mostly benign tumors that develop from meningothelial cells of the arachnoid mater [1–4]. Intracranial meningiomas are the most prevalent primary brain tumors [1,5,6]. The Central Brain Tumor Registry of the United States (CBTRUS) reports that meningiomas account for over 33% of all primary brain tumors, with an incidence rate of 8.05 per 100,000 person-years [5]. In Palestine, meningiomas constituted around 42.8% of the non-malignant primary brain tumors among adults and adolescents, with a total incidence rate of 1.02 per 100,000 person-years [7]. They are more often seen in females than in males [4,8,9]. A total of 15 meningioma subtypes were identified in the WHO Classification of CNS Tumors [10]. The 2021 WHO Classification of CNS Tumors considers meningioma, with its 15 morphological subtypes, as a single type [11]. These 15 subtypes are classified into three different WHO grades: grade I meningiomas, atypical meningioma (grade II), and anaplastic (malignant) meningioma (grade III), based on their behavior [4,10,11].

In the Middle East, epidemiological studies on meningiomas remain limited but provide valuable comparisons. A recent study from Saudi Arabia reported that meningiomas constituted 15% of primary brain tumors [12]. In Lebanon, Meningiomas represented approximately 29.6% of histopathological primary brain tumors [13]. Reported data from Syria between the years 2002–2008 showed that meningiomas account for 1.1% of child brain tumors [14]. A study conducted in Jordan in 2015 reported that meningiomas were the most common histological types, which account for 26.2% of primary brain tumors [15].

Numerous risk factors can contribute to the development of meningioma. Exposure to ionizing radiation is a risk factor for meningioma [9,16]. A family history of meningioma in first-degree relatives likely increases the risk for meningioma [9,17]. Anthropometric factors such as increasing height and BMI in women were found to be associated with an increased risk for meningioma [18]. The association between head trauma and cell phone use with meningioma is not yet well-established [4].

The clinical presentation of meningiomas is dependent on their anatomical location. There is no specific pathognomonic presentation of meningioma. However, if symptomatic, meningiomas usually present with headache, seizures, or focal neurological symptoms as a result of compression or invasion of adjacent structures [1, 6]. While biopsy and histopathology are considered the definitive methods for diagnosing meningioma, MRI is the most commonly used imaging modality, and it usually demonstrates a homogeneously enhancing extra-axial mass [6]. Surgical resection is often the mainstay of treatment of symptomatic meningioma and is mostly curative [1,6,8,19]. Due to the risk of recurrence and mortality, adjunctive radiotherapy is reserved for meningioma grade II and III [1,6]. Although mostly benign, meningiomas can cause significant morbidity, either through symptoms associated with their natural history or the complications of the treatments used [2,20]. The risk of mortality increases with higher grade [6].

Although the incidence of meningiomas has been rising worldwide [4], epidemiological data on meningiomas in Palestine remain limited. Understanding the burden and characteristics of meningioma in Palestine is essential for enhancing patient outcomes and tailoring healthcare resources. This study aims to provide insights into the epidemiological characteristics of meningioma in the Palestinian population between 2018 and 2023, with potential implications for future research and healthcare policy. In the absence of a centralized national cancer registry in Palestine, referral-center–based datasets provide valuable, though inherently limited, insights into disease burden and care patterns.

## 2 Materials and methods

### 2.1 Study design and setting

This study was a retrospective chart review study to describe the epidemiology of intracranial meningioma in Palestine. It was conducted at Al-Makassed Hospital in Jerusalem, the largest referral center for primary brain tumors. While this represents a large proportion of meningioma cases, it may not be representative of all cases nationally, as some cases were treated in other facilities or referred abroad. Therefore, this study may provide the best hospital-based estimate of the incidence of intracranial meningiomas in Palestine, rather than the accurate incidence rate. The targeted population consisted of Palestinians diagnosed with intracranial meningioma at Al-Makassed Hospital or referred to the hospital for treatment of intracranial meningioma during the period from 2018 to 2023. Cases of intracranial meningioma were identified based on the electronic health records at Al-Makassid Hospital according to the following inclusion criteria: (1) patients who presented with clinical signs and symptoms of intracranial meningioma (such as headache, focal neurological deficit, seizures, etc.), (2) had a radiological diagnosis of intracranial meningioma by Brain MRI, and (3) had a confirmed histopathological diagnosis of intracranial meningioma. Accordingly, patients with incidentally identified meningiomas on radiological studies or those treated at other hospitals were excluded. Moreover, patients with spinal meningiomas, as well as those lacking histopathology reports or MRI reports, were excluded from the study. The study size was determined by the total number of eligible cases during the study period.

### 2.2 Data collection

Data were collected from patients’ clinical, histopathological, and MRI reports between July 9, 2023, and November 16, 2023. A Google Form was created to facilitate data collection, comprising two main sections: the first included sociodemographic characteristics such as age at diagnosis, sex, type of residency, and place of residency; the second covered clinical data, including year at the first diagnosis, histopathological type, tumor behavior, laterality, initial symptoms, management type, and mortality. Histopathological types of meningioma were coded according to the 2016 WHO Classification for CNS tumors [10]. This version was chosen over the 2021 version because the patient data were documented in reports based on the 2016 classification, and advanced molecular techniques required for the 2021 version were not available. Data on meningioma grade were available for all cases; however, subtype data were missing in 62% of the histopathological reports. All cases with missing subtype information were classified as grade I meningiomas; no cases were missing subtype information for grade II or III meningiomas. In many cases, missing data on the subtype likely resulted from information bias stemming from limited or incomplete documentation rather than from systematic error. Potential confounding factors were considered conceptually, including referral patterns related to the hospital’s role as a tertiary referral center, differential access to healthcare services across geographic regions, and variations in diagnostic availability over time; however, adjustment for these factors was not feasible due to the descriptive nature of the study and limitations of available data.

The referral hospital-based minimum estimated annual incidence rates were calculated using mid-year population estimates obtained from the Palestinian Central Bureau of Statistics (PCBS) as denominators; however, these estimates should be interpreted as hospital-based incidence proxies rather than population-based rates, given the single-center study design. Population estimates for 2023 were obtained by extrapolating the mean annual population increase calculated from national data for 2018–2022 (total population increase divided by four years), assuming a stable linear growth trend. The same method was applied separately to male and female populations. All incidence rates were calculated with exact Poisson 95% confidence intervals. All-cause mortality was defined as death occurring within 30 days following surgery, as documented in hospital records.

### 2.3 Data analysis

The statistical analysis software, Statistical Package for Social Sciences (SPSS), version 26.0, was used for data analysis. The data was organised and coded in a Google Spreadsheet, and then transferred to SPSS. A basic descriptive analysis, including means, standard deviations, frequency, and percentages, was performed for each categorical variable. Cross-tabulation and Fisher’s Exact tests were used to compare the categorical variables. The significance level was first set at 5%, and p-values below 0.05 were considered statistically significant. However, to account for multiple hypothesis testing, the Bonferroni correction was applied across a family of nine independent statistical comparisons, including analyses examining associations between WHO tumor grade, management approach, mortality, and sociodemographic variables (Tables 4, 7, and 8). Accordingly, the significance threshold was adjusted to α = 0.05/9 ≈ 0.005. Only associations with adjusted p-values below this threshold were considered statistically significant. Due to the risk of model overfitting from the low number of mortality events (n = 11), using a binary logistic regression for mortality analysis was not feasible. Missing data on the subtype of meningioma were labelled as ‘Missing Subtype’ in the analysis of histopathological subtype proportions and crude incidence rate estimates, to assess whether the missing data were more common in a specific sex compared to the other. The cases of meningioma with missing data on the tumor’s laterality were not included in the analysis. No sensitivity analyses were performed.

### 2.4 Ethical consideration

The Institutional Review Board (IRB) at An-Najah National University (IRB Ref: Med. June. 2023/12) and the Palestinian Ministry of Health granted ethical approval to conduct the study. The IRB waived the informed consent for this retrospective study. During this study, no patients’ personal identifiers were collected. All patients’ records were returned to Al-Makassed Hospital after the end of the study.

## 3 Results

### 3.1 Sociodemographic characteristics of the patients

A total of 204 patients were identified and included in this study. The mean age at diagnosis was 51.12 (±13.96) years. Two-thirds of the patients were females (66.7%). Approximately 48% of the patients resided in rural areas, 44.1% in urban areas, and around 8% in refugee camps. The majority of the patients were residents of the West Bank (60.8%). The sociodemographic characteristics of the patients are summarised in Table 1.

**Table 1:** Sociodemographic characteristics of the patients.

Variable	Mean (SD)/Frequency (%)
**Age:**	51.12 (±13.96)
Male	68 (33.3)
Female	136 (66.7)
City	90 (44.1)
Village	98 (48.0)
Refugee Camp	16 (7.9)
West Bank	124 (60.8)
Gaza Strip	80 (39.2)
**Total**	**204 (100)**

### 3.2 Incidence rate of meningiomas in Palestine during the period from 2018 to 2023

**Fig 1:**
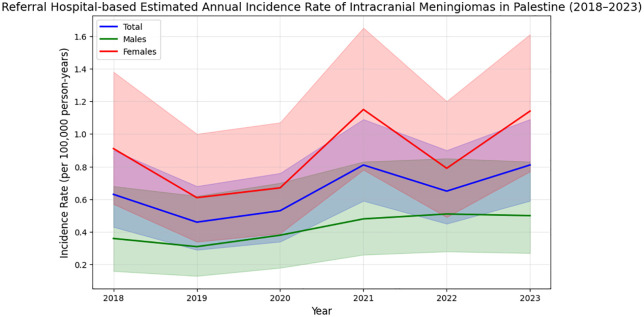
Annual hospital-based incidence rates of intracranial meningioma per 100,000 population from 2018 to 2023. (The shaded areas represent 95% confidence intervals. Rates were calculated using the number of newly diagnosed intracranial meningioma cases treated at Al-Makassed Islamic Charitable Hospital as the numerator and mid-year population estimates from the Palestinian Central Bureau of Statistics (PCBS) as denominators. These estimates should be interpreted as hospital-based incidence proxies rather than population-based incidence rates) and Table 2 illustrate the annual incidence rate in the total population, males, and females.

The estimated average annual crude incidence rate of meningioma was 0.65 per 100,000 person-year (95% CI: 0.56–0.75). The annual incidence rate of meningioma has increased from 2018 to 2023, with the highest value in 2021. The average annual incidence rate of meningioma in females was higher than in males (0.88 vs 0.43 per 100,000 person-years), with an incidence rate ratio (IRR) of 2.07 (95% CI: 1.53–2.81; p = 0.003) (Fig 1).

**Table 2:** Referral Hospital-based Estimated Annual Incidence Rate of Meningioma in Palestine based on data from 2018 to 2023.

Year	Cases	Population	IR*	(95% CI)	Males	Males IR*	(95% CI)	Females	Females IR*	(95% CI)
2018	31	4,915,349	0.63	(0.43-0.90)	2,500,064	0.36	(0.16-0.68)	2,415,285	0.91	(0.57-1.38)
2019	23	5,038,918	0.46	(0.29-0.68)	2,562,304	0.31	(0.13-0.62)	2,476,614	0.61	(0.34-1.00)
2020	27	5,164,640	0.53	(0.34-0.76)	2,625,710	0.38	(0.18-0.70)	2,538,930	0.67	(0.39-1.07)
2021	43	5,290,925	0.81	(0.59-1.09)	2,689,117	0.48	(0.26-0.83)	2,601,808	1.15	(0.78-1.65)
2022	35	5,419,053	0.65	(0.45-0.90)	2,753,535	0.51	(0.28-0.85)	2,665,518	0.79	(0.49-1.20)
2023	45	5,548,457	0.81	(0.59-1.09)	2,818,549	0.50	(0.27-0.83)	2,729,908	1.14	(0.77-1.61)
**Total**	204	Average annual IR*	0.65	(0.56-0.75)	Average annual IR*	0.43	(0.33-0.54)	Average annual IR*	0.88	(0.74-1.04)

* Crude Incidence rate per 100,000 person-year.

CI: Confidence Interval, calculated by pooling cases and person-years across six years and using the exact Poisson 95% CI.

Note: These are referral hospital-based estimated annual incidence rates for Meningioma and do not reflect accurate population-based Incidence rates.

### 3.3 Histopathological subtypes of meningioma

Approximately 62.2% of the reports were missing the subtype of meningioma. The grade of these meningiomas was reported without the exact subtype. Among cases with available subtype data, anaplastic and rhabdoid meningiomas were infrequently observed. One case of rhabdoid meningioma was found in a male patient. Anaplastic (malignant) meningioma was more common in males compared to females (75% vs 25%). The histopathological subtypes of meningioma and their average incidence rates are summarised in Table 3.

**Table 3:** Histopathological Subtypes of Meningioma and their average IR (62% of data are missing).

Grade I										
Subtype ^b^	Code	Proportion			Estimated Crude IR*					
		Total (%)	Females (%)	Males (%)	Total	95% CI^a^	Females	95% CI^a^	Males	95% CI^a^
Unspecified subtypes	–	127 (62.2)	91 (71.65)	36 (28.35)	–	–	–	–	–	–
Meningiothelial meningioma	9530/1	5 (2.5)	5 (100)	0 (0)	0.02	(0.01-0.04)	0.03	(0.01-0.08)	0	(0-0.02)
Fibrous meningioma	9535/1	5 (2.5)	3 (60)	2 (40)	0.02	(0.01-0.04)	0.02	(0-0.06)	0.01	(0-0.05)
Transitional meningioma	9538/1	7 (3.4)	2 (28.57)	5 (71.43)	0.02	(0.01-0.05)	0.01	(0-0.05)	0.03	(0.01-0.07)
Psammomatous meningioma	9530/1	8 (3.9)	7 (87.5)	1 (12.5)	0.03	(0.01-0.05)	0.05	(0.02-0.09)	0.01	(0-0.03)
Angiomatous meningioma	9537/1	3 (1.5)	2 (66.67)	1 (33.33)	0.01	(0-0.03)	0.01	(0-0.05)	0.01	(0-0.03)
Microcystic meningioma	9532/1	3 (1.5)	3 (100)	0 (0)	0.01	(0-0.03)	0.02	(0-0.06)	0	(0-0.02)
Secretory meningioma	9530/0	5 (2.5)	3 (60)	3 (40)	0.02	(0.01-0.04)	0.02	(0-0.06)	0.01	(0-0.05)
Lymphoplasmacyte-rich meningioma	9537/1	3 (1.5)	3 (100)	0 (0)	0.01	(0-0.03)	0.02	(0-0.06)	0	(0-0.02)
Total	–	166 (81.4)	119 (71.7)	47 (28.3)	0.53	(0.45-0.62)	0.77	(0.64-0.92)	0.29	(0.22-0.39)
**Grade II**										
**Subtype**	**Code**	**Proportion**			Estimated Crude IR*					
		**Total (%)**	**Females (%)**	**Males (%)**	**Total**	**95% CI** ^a^	**Females**	**95% CI** ^a^	**Males**	**95% CI** ^a^
Atypical meningioma	9539/1	33 (16.2)	16 (48.48)	17 (51.52)	0.11	(0.07-0.15)	0.1	(0.06-0.17)	0.11	(0.06-0.17)
Total	–	33 (16.2)	16 (48.48)	17 (51.52)	0.11	(0.07-0.15)	0.1	(0.06-0.17)	0.11	(0.06-0.17)
**Grade III**										
**Subtype**	**Code**	**Proportion**			Estimated Crude IR*					
		**Total (%)**	**Females (%)**	**Males (%)**	**Total**	**95% CI** ^a^	**Females**	**95% CI** ^a^	**Males**	**95% CI** ^a^
Rhabdoid meningioma	9538/3	1 (0.5)	0 (0)	1 (100)	0.003	(0-0.02)	0	(0-0.02)	0.01	(0-0.03)
Anaplastic (malignant) meningioma	9531/3	4 (1.9)	1 (25)	3 (75)	0.01	(0-0.03)	0.01	(0-0.04)	0.02	(0-0.05)
Total	–	5 (2.4)	1 (20)	4 (80)	0.02	(0.01-0.04)	0.01	(0-0.04)	0.03	(0.01-0.06)

*Estimated Crude Incidence Rate per 100,000 person-year.

^a^CI: Confidence Interval, calculated by pooling cases and person-years across six years and using the exact Poisson 95% CI.

^b^Missing data explanation: Data on the grade of the meningioma were available; however, the data on the subtype were missing in 62% of the histopathological reports of grade I meningioma. Data regarding the subtypes of Grade II and III were complete.

Note: Subtype frequencies should not be interpreted as representative of the overall distribution due to substantial missing data.

### 3.4 The WHO grades of meningiomas

Meningiomas among the Palestinian patients were mostly grade I (benign) (81.4%). Benign meningiomas were more common in females than males (87.5% vs 69.1%, p-value 0.003), and grade III malignant meningiomas were more common in males (5.9% vs 0.7%, p-value 0.003). The type and place of residence were not associated with the grade of meningiomas (Table 4).

**Table 4:** The WHO grades of Meningiomas in relation to other variables.

Variable	WHO grades of Meningiomas			p-value^a^
	Grade I (%)	Grade II (%)	Grade III (%)	
**Gender:**				
Male	47 (69.1)	17 (25)	4 (5.9)	0.003*
Female	119 (87.5)	16 (11.8)	1 (0.7)	
**Type of Residency:**				
City	73 (81.1)	15 (16.7)	2 (2.2)	0.629
Village	79 (80.6)	17 (17.3)	2 (2.1)	
Refugee Camp	14(87.5)	1 (6.25)	1 (6.25)	
**Place of Residency:**				
West Bank	98 (79)	22 (17.7)	4 (3.3)	0.518
Gaza Strip	68 (85)	11 (13.75)	1 (1.25)	
**Total**	**166 (81.4)**	**33 (16.2)**	**5 (2.4)**	

* Statistically significant.

^a^Fisher’s Exact test.

### 3.5 Laterality of the Meningioma in relation to the brain hemispheres

Data on the laterality of meningiomas relative to the brain hemispheres were reported in 179 patients, with 12.25% missing data. 39.1% of the meningiomas were located in the right hemisphere, 33% in the left hemisphere, and 27.9% crossed the midline. Table 5 summarises the laterality of meningiomas.

**Table 5:** Laterality of the Meningioma in relation to the brain hemispheres.

Location	Frequency	Percentage (%)
Right	70	39.1
Left	59	33
Crossing the Midline	50	27.9
**Total**	179*	

* A total of 25 records with missing data.

### 3.6 First presenting sign or symptom in meningioma

Focal signs (motor or sensory signs), headaches, and seizures were the most commonly reported presenting symptoms (39.7%, 36.8%, and 9.8%, respectively). Cognitive and emotional dysfunction, nausea/vomiting/dizziness, and neurogenic bladder were the least commonly reported presenting symptoms (1.5%, 1%, and 0.5%, respectively). The first presenting signs or symptoms are summarised in Table 6.

**Table 6:** First Presenting Sign or Symptom in Meningioma.

First sign/symptom	Frequency	Percentage (%)
Focal signs (motor or sensory signs)	81	39.7
Headache	75	36.8
Seizure	20	9.8
Mental status alteration (drowsiness, confusion, etc)	8	3.9
Cognitive and emotional dysfunction	3	1.5
Nausea/vomiting/dizziness	14	6.9
Dysarthria	2	1
Neurogenic bladder/bowel	1	0.5

### 3.7 Type of management in meningioma

The most common management was surgical resection (96.6%), primarily via open craniotomy and Simpson Grade I Gross Total Resection (GTR). Malignant meningiomas (grade III) were mostly managed by surgical resection alone rather than surgical resection followed by radiotherapy (80% vs 20%); however, this result was not statistically significant (p-value 0.02 > 0.005). Only 3% of grade II meningiomas were managed with surgical resection and chemotherapy. The management of meningioma by grade is summarized in Table 7.

**Table 7:** Type of Management in Meningioma.

Type of Management	n (%)	Grade I (%)	Grade II (%)	Grade III (%)	p-value*
Surgical resection (Simpson grade I GTR)	197 (96.6)	163 (98.2)	30 (91)	4 (80)	0.02
Surgical resection and Radiotherapy	6 (3)	3 (1.8)	2 (6)	1 (20)	
Surgical resection and Chemotherapy	1 (0.4)	0 (0)	1 (3)	0 (0)	
**Total**	**204**	**166 (81.4)**	**33 (16.2)**	**5 (2.4)**	

* Fisher’s Exact test.

*GTR: Gross Total Resection.*

### 3.8 All-cause mortality within 30 days following surgery

The all-cause mortality rate within 30 days following surgery was 5.4%. The mortality rate was higher among female patients, patients with benign meningiomas, patients managed with surgery alone, patients living in urban areas, and patients from the Gaza Strip, but the results were not statistically significant (p-value = 0.343, 0.770, 1.0, 0.235, and 0.115, respectively). The all-cause 30-day mortality rates in meningioma in relation to these variables are summarised in Table 8.

**Table 8:** All-cause Mortality within 30 Days following surgery in patients with Meningioma.

Variable	Total (n)	Deceased (%)	Alive (%)	p-value*
**Gender:**				
Male	68	2 (3)	66 (97)	0.343
Female	136	9 (6.6)	127 (93.4)	
**Grade of Meningioma:**				
Grade I	166	10 (6)	156 (94)	0.770
Grade II	33	1 (3)	32 (97)	
Grade III	5	0 (0)	5 (100)	
**Type of Management:**				
Surgical resection alone	197	11 (5.6)	186 (94.4)	1.0
Surgical resection and Radiotherapy	6	0 (0)	6 (100)	
Surgical resection and Chemotherapy	1	0 (0)	1 (100)	
**Type of Residency:**				
City	90	7 (7.8)	83 (92.2)	0.253
Village	98	3 (3)	95 (97)	
Refugee Camp	16	1 (6.25)	15 (93.75)	
**Place of Residency:**				
West Bank	124	4 (2)	120 (96.8)	0.115
Gaza Strip	80	7 (8.7)	73 (91.3)	
**Total**	**204**	**11 (5.4)**	**193 (94.6)**	

* Fisher’s Exact test.

## 4 Discussion

The worldwide incidence of primary brain tumors, including meningiomas, is increasing, possibly due to improved access to healthcare, increased life expectancy, and the usage of advanced imaging modalities [21]. This study analyzed the epidemiological characteristics of meningioma in Palestine. This study analyzed the reported data of patients with meningioma in Palestine. It provides insights into the epidemiological characteristics of meningioma among the Palestinian population.

### 4.1 Intracranial meningioma incidence in Palestine

The hospital-based minimum estimated crude incidence rate of meningioma in Palestine was 0.65 per 100,000 person-years. The temporal trend analysis of the estimated annual incidence rate showed that meningioma incidence increased from 2018 to 2023. This temporal trend should be interpreted cautiously, as it may reflect increased referral volumes and improved availability of diagnostic imaging techniques in recent years; the apparent increase likely reflects improved detection rather than a biological rise [21]. Further studies are needed to examine the temporal trends in intracranial meningiomas.

In general, the estimated minimum incidence rate of intracranial meningioma in Palestine was low; however, it reflects a hospital-based minimum estimate and is not directly comparable to population-based registry data. The lack of a national population-based registry in Palestine prevents the direct comparison of the estimated incidence rate with population-based registry data. For example, well-established registry data showed that the incidence rate of meningioma in the Netherlands is 4.69 per 100,000 person-years, and in the United States, 8.05 per 100,000 person-years [5,22,23]. Epidemiological data on meningiomas in the Middle East are scarce but provide valuable regional comparisons. In Saudi Arabia, meningiomas accounted for 15% of primary brain tumors [12], while in Lebanon they represented 29.6% [13]. In Jordan, a 2015 study reported meningiomas as the most frequent histological subtype, representing 26.2% of primary brain tumors [15]. However, given the differences in methodology between this study and those conducted in other countries, this result should be interpreted with caution. Observed differences are more likely to be explained by healthcare access, referral patterns, and demographic structure than by true geographic variation in disease biology.

Additionally, the low hospital-based estimated incidence of meningioma may be influenced by challenges related to healthcare resources and patients’ access to the main referral center for meningioma. The limited ability of patients with suspected meningioma to access the tertiary centers, and even the ability to perform CT/MRI scans, as well as the younger demographic population in Palestine compared with the Netherlands and the United States, have contributed to the lower estimated incidence in the country. Moreover, the absence of a centralized national cancer or neurosurgery registry limits our ability to provide precise population-based incidence rates. Therefore, our findings should be interpreted as hospital-based estimates. Establishing a national registry would be essential for accurate epidemiological characterization of meningiomas in Palestine.

Given that our study specifically focused on intracranial meningiomas, this may limit comparability with studies that report all meningiomas, including spinal cases. The exclusion of spinal meningiomas may explain some differences with the literature that reports all meningioma cases combined. Therefore, comparisons of meningioma incidence rates should be interpreted with caution.

### 4.2 Incidence rate of intracranial meningioma in relation to sex

In the present study, the estimated incidence of meningioma was higher in females than in males, consistent with other studies conducted in Saudi Arabia, the Netherlands, the United States, Southern Japan, and India [5,22–26]. Although sex-related differences in meningioma incidence have been widely documented, the current study did not evaluate individual-level hormonal, genetic, or environmental risk factors. Therefore, no inferences can be made regarding the underlying biological mechanisms contributing to this observed disparity. The higher incidence observed among females should be interpreted within an epidemiological context and may reflect multifactorial influences described in the literature [27]. Future population-based studies incorporating detailed clinical, hormonal, and molecular data are needed to further elucidate the determinants of sex differences in meningioma incidence.

### 4.3 Histopathological subtypes of intracranial meningioma

In the present study, a reliable assessment of the distribution of histopathological subtypes of intracranial meningioma was not possible. Although the WHO grade was available for all cases, subtype information was missing for a substantial proportion of Grade I tumors (62%), which were recorded in the pathology reports as meningioma without further specification. As a result, subtype-level analyses were limited to cases with explicitly documented histopathological classifications and should not be interpreted as representative of the overall subtype distribution among Palestinian patients. Grade I (benign) meningiomas constituted the majority of cases in this cohort, followed by Grade II and Grade III tumors. Higher-grade meningiomas were relatively more frequent among male patients, a finding that aligns with previous studies suggesting sex-related differences in tumor aggressiveness [9,28–30].

However, given the extent of missing subtype data in Grade I tumors, no conclusions can be drawn about the relative frequency or predominance of specific histopathological subtypes in this population. The high proportion of unspecified Grade I subtypes likely reflects limitations in routine pathological reporting and resource constraints rather than a true epidemiological pattern. Improving the completeness and standardization of histopathological documentation, as well as access to advanced diagnostic techniques, would be essential for accurately characterizing meningioma subtypes in future studies and for enabling meaningful comparisons with regional and international data.

### 4.4 Tumor laterality in relation to cerebral hemispheres

The laterality of meningioma was evaluated in this study. Meningiomas occurred slightly more in the right brain hemisphere (39.1%) than in the left brain hemisphere (33%), and 27.9% of the meningiomas crossed the midline; however, this result was not significant. No specific predilection of meningioma to develop in one brain hemisphere compared to the other was identified in this study. Similar results were reported in a previous study [31].

### 4.5 Clinical signs and symptoms at presentation of intracranial meningioma

At presentation, most of the patients in this study had focal neurological symptoms (FNS), both motor and sensory (39.7%), followed by headache (36.8%), and seizures (9.8%). According to a study by *Louis et al*. conducted in 2016, the majority of meningioma patients presented with FNS, and another study also indicated headache as a common first presentation for patients with meningioma [10,32]. Intracranial meningioma has the potential to arise in different locations, enabling it to compress the adjacent neural tissues, leading to focal neurological deficits.

### 4.6 Management of intracranial meningioma in Palestine

In this study, meningiomas were mostly managed by surgical resection with Simpson grade I GTR (96.6%), rather than surgery with radiotherapy (3%) or chemotherapy (0.6%). Notably, the surgical resection alone modality remains predominant even in higher-grade meningiomas (grade III), after which surgery and radiotherapy are used [33]. Several studies have shown the benefits of concomitant use of chemotherapy or radiotherapy along with surgery in aggressive or recurrent tumors [34,35]. The predominance of surgical resection alone, even for higher-grade tumors, likely reflects the limited availability of radiotherapy and chemotherapy in Palestine, highlighting a gap compared to international guidelines. The limited availability of adjuvant therapy for intracranial meningioma remains a challenge for the healthcare system in resource-limited settings, such as in Palestine. This may lead to worse outcomes than in well-developed, well-equipped settings, where international management guidelines are adhered to. It is worth mentioning that patients managed with surgery alone have a higher all-cause mortality rate (5.6%) than patients managed with concomitant therapies. Although this result is not statistically significant, it is consistent with other studies [36].

### 4.7 All-cause mortality rate within 30 days following surgery in patients with intracranial meningioma in Palestine

The results of this study show an all-cause 30-day mortality rate of 5.4%, with certain demographic factors contributing. Surprisingly, grade I meningioma had a higher mortality rate than grade II/III; however, this result was statistically insignificant. This finding may be explained by the small number of grade II/III tumors (n = 38), which may have reduced power to detect mortality differences. Additionally, this finding may result from the more aggressive management of grade II/III tumors, which could potentially lower mortality in this category of patients. Overall, further studies with larger samples are needed to examine differences in mortality by intracranial meningioma grade.

### 4.8 Study limitations

The study has some limitations. First, considering the retrospective nature of the study, controlling information bias and limitations in documentation were challenging. The main limitation would be the accessibility of diagnostic resources for patients, as Al-Makassed Hospital is considered the main referral center for meningioma diagnosis and treatment. Patients from Gaza as well as from the West Bank cannot always access the hospital to get diagnosed and treated. Additionally, we cannot confirm the proportion of all meningioma cases represented in this study. Therefore, the incidence estimates provided in this study are based on a major referral. Our incidence estimates should be interpreted as minimum hospital-based estimates, which are likely to underestimate the true national incidence. Although Bonferroni correction was applied to reduce the risk of false-positive findings, this conservative approach may have increased the risk of type II error, particularly given the limited sample size and low number of mortality events.

While multivariate analysis for mortality using binary logistic regression would be more appropriate, it was not feasible due to the small number of events (n = 11). Thus, mortality results should be interpreted with caution. Moreover, during data collection, information regarding the subtype of 62% of meningioma cases was missing from the patients’ records. This large proportion of missing subtype data precluded a reliable estimation of the most common histopathological subtype in Palestine. This disproportionately affected our ability to compare subtype distributions with regional and global data. Incomplete documentation in some medical records and limited access to diagnostic resources were likely the cause of missing data, rather than systematic exclusion of cases.

Enhancing the documentation process would greatly improve the availability of relevant data on meningioma subtypes and other primary brain tumors. Further studies are recommended to monitor the changes in the epidemiology of meningioma in Palestine and identify the most common meningioma subtype among the Palestinian population. Finally, although the 2021 WHO classification incorporates molecular markers, these were not consistently available in our setting, making uniform reclassification impossible. This limitation should be taken into consideration when comparing with more recent studies.

### 4.9 Recommendations and clinical implications

The findings of this study carry important clinical implications for the management of intracranial meningiomas in Palestine. The predominance of surgery as the sole management approach highlights a significant gap in the availability of radiotherapy and chemotherapy, which are essential components of management for higher-grade tumors and for patients who are poor surgical candidates. Limited access to these modalities may compromise patient outcomes, underscoring the need to expand oncological services and infrastructure. The observed female predominance and increasing detection trends emphasize the importance of early diagnosis and resource planning to meet the expected rise in demand for neurosurgical and oncological care. Establishing a national cancer registry and improving access to advanced diagnostic and treatment facilities would enable more comprehensive care, align local practices with international standards, and ultimately reduce the disease burden.

## 5 Conclusion

This study is the first to provide insights into the epidemiological characteristics of intracranial meningioma in Palestine. While the minimum estimated incidence of intracranial meningioma in Palestine is low, it remains common and contributes to considerable morbidity, placing a strain on the healthcare system. The low estimated incidence of meningioma in Palestine can be attributed to several factors, including the retrospective nature of this study, resource limitations, and restricted patient access to the major referral center for brain tumors. We found a female predominance and a trend toward increasing detection in recent years. No specific predilection for meningioma to develop in any brain hemisphere was found in this study. This study was unable to identify the most common subtype of intracranial meningioma in Palestine due to incomplete subtype data, underscoring the need for further research. Surgical resection alone was the most common management approach, reflecting limited access to radiotherapy and chemotherapy. All-cause mortality rate was low overall but should be interpreted cautiously given the small number of deaths and limited follow-up data. These findings highlight the need for a national cancer registry and expanded treatment resources to improve care for patients with intracranial meningioma in Palestine.

## Supporting information

S1 Raw Data Meningioma epidemiology. (XLSX)
